# Anesthetic Management of a Patient With Juvenile Hyaline Fibromatosis: A Case Report Written With the Assistance of the Large Language Model ChatGPT

**DOI:** 10.7759/cureus.35946

**Published:** 2023-03-09

**Authors:** Scott Segal, Ashish K Khanna

**Affiliations:** 1 Anesthesiology, Wake Forest School of Medicine, Winston-Salem, USA; 2 Anesthesiology, Wake Forest School of Medicine, Winston Salem, USA

**Keywords:** chatgpt, large language model, hyaline fibromatosis syndrome, anesthetic management, juvenile hyaline fibromatosis

## Abstract

This case report was written with the assistance of the large language model known as ChatGPT, a form of generative artificial intelligence that can write grammatically correct and semantically meaningful prose on a multitude of topics. Here, it has assisted us in presenting a case of anesthetic management for a case of Juvenile Hyaline Fibromatosis (JHF), an extremely rare genetic disorder that is part of a spectrum of diseases presently characterized as Hyaline Fibromatosis Syndrome (HFS), which also includes a more severe variant presenting in infancy. HFS is caused by autosomal recessive mutations in the ANTXR2 (anthrax toxin receptor cell adhesion molecule 2) gene, which binds collagen IV and laminin, suggesting that it may be involved in extracellular matrix adhesion. Defects in this molecule lead to abnormal deposition of hyaline material in perivascular areas, presenting as cutaneous lesions, joint contractures, and in some cases internal organ dysfunction. Anesthetic management of patients with JHF may present difficulties with patient positioning and airway management. Most reports of anesthetic management concern children with severe disease and adult reports are uncommon. We present a case of JHF in a 39-year-old woman managed for resection of a lower extremity cutaneous lesion. The anesthetic management of this relatively minor case was uneventful, but the process of drafting this report with the assistance of the new software tool ChatGPT was informative of both its strengths and limitations.

## Introduction

Juvenile hyaline fibromatosis (JHF) is a rare genetic disorder characterized by the accumulation of hyaline material in the body's tissues. The disease is caused by mutations in the ANTXR2 (anthrax toxin receptor cell adhesion molecule 2) gene and affects multiple systems, including the skin, bones, and internal organs. JHF is a progressive disorder and there is currently no cure. It is an extremely rare disorder, with only a few hundred cases reported worldwide.

The disease affects males and females equally and has a global distribution. Clinical manifestations of JHF can range from mild to severe and often require extensive medical and surgical management [1].

Anesthetic care for patients with JHF can be challenging due to the involvement of multiple systems and the progressive nature of the disease. In this case report, we present the anesthetic management of a patient with JHF undergoing surgery, highlighting the importance of a thorough preoperative evaluation and a multidisciplinary approach to ensure safe and effective anesthesia care [2-4]. 

Our patient underwent a minor procedure and had a benign anesthetic course, we elected to report this case for two reasons. First, there are few published case reports of which we are aware of adults with the syndrome undergoing surgical procedures. More importantly, this report was written with the assistance of ChatGPT, a large language model type of generative artificial intelligence. Our report highlights the potential as well as the limitations of this technology.

## Case presentation

**Figure 1:**
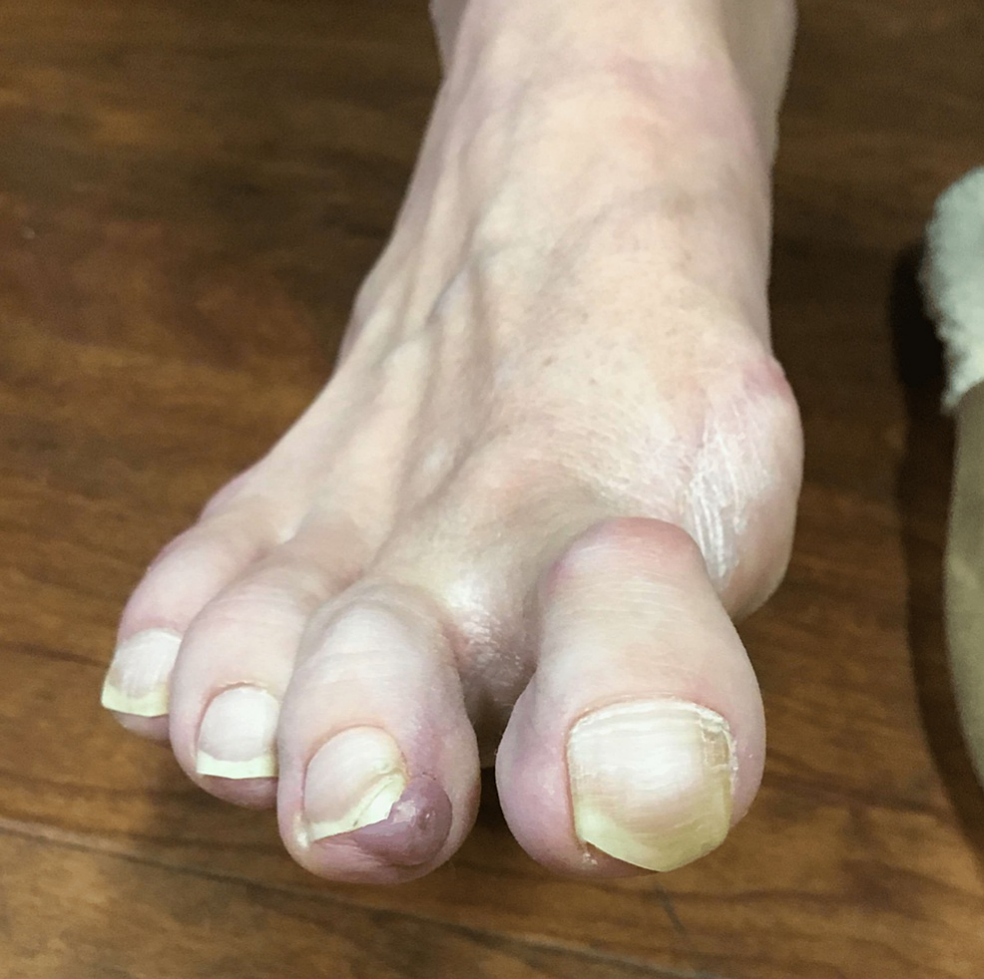
The patient’s lesion preoperatively

The patient is a 39-year-old female who presented for resection of a benign lesion on the second toe of the right foot (Figure 1). The patient consented to this report and participated actively in its preparation and review.

**Figure 2:**
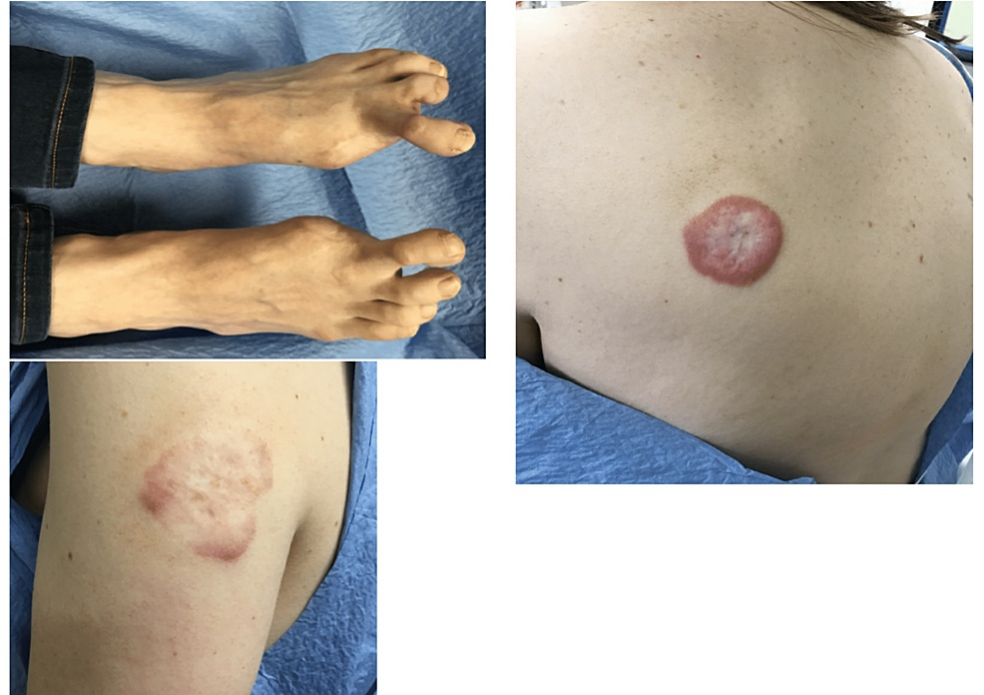
Previous resections of the great toes and lesions on the left arm and back

She had a long history of JHF, followed for several years by a dermatologist, and for approximately 25 years prior to surgery by plastic surgeons. She first presented in her early teens with a persistent lesion on her right knee which recurred multiple times after resection and/or steroid injection. After multiple rounds of treatment, she developed persistent and bothersome lesions on her great toes, which also recurred after resections. She noted that the fibromas had so enlarged her toes that she was unable to wear ordinary shoes. She was referred to plastic surgery and underwent more extensive resection efforts including skin grafting, but experienced recurrence and eventually underwent bilateral great toe amputation. She was also followed for persistent lesions on her left arm and upper back (Figure 2).

Over time, she decided to forego additional resection attempts on her knee and the lesion gradually receded. She has made the same decision regarding her arm and back, with substantial regression of the arm lesion, but the persistent lesion on her right toe led her to seek limited resection for the present operation.

Her past medical history was otherwise unremarkable, notably only for a benign splenic cyst, a bicornuate uterus, allergic rhinitis, and a history of keloid formation after an unrelated surgery. She did not smoke or use alcohol. She had no drug allergies and did not regularly use prescription medications. The review of systems was otherwise negative. She had no history of problems with previous anesthetics. She has no children and knows of no affected relatives. She had not undergone genetic testing.

On examination, the lungs were clear, and the heart showed regular rate and rhythm without murmurs. BP was 110/58 mmHg, HR 80/minute, SpO_2_ 98% on room air. Examination of the airway revealed 3-fingerbreadth mouth opening, 4-fingerbreadth thyromental distance, Mallampati class I oropharyngeal features, full range of motion of the neck, normal gingiva and teeth, and otherwise normal facies. Laboratory studies (electrolytes, liver function tests, TSH, CBC) obtained less than three months prior to the operation were unremarkable, and additional preoperative studies were not performed given the nature of the procedure.

A 20 G peripheral IV was placed. Standard ASA monitors were applied. The patient received 2 mg of midazolam for preoperative anxiolysis. General anesthesia was selected due to the brief outpatient nature of the procedure and patient preference. Anesthesia was induced with 250 mg propofol and a supraglottic airway (laryngeal mask airway) was positioned on the first attempt without difficulty. Anesthesia was maintained with isoflurane, 1.3%-1.6% end-tidal concentration; the patient breathed spontaneously during most of the case but was supplemented with SIMV and pressure support prior to surgical incision. She received glycopyrrolate for its antisialagogue effect, postoperative nausea and vomiting prophylaxis with ondansetron, and pre-incision cefazolin. Her anesthetic course was uneventful and she emerged uneventfully and the LMA was removed. She was taken to the outpatient post-anesthesia care unit where she rapidly recovered and was discharged home.

## Discussion

The patient presented in this case report with JHF underwent an uneventful anesthetic management for a toe lesion resection. The clinical course of her disease had been relatively benign, with only diffuse skin involvement noted, and thus her care did not pose any major difficulties. However, the presentation of JHF is quite variable and can have important anesthetic implications [2-10]. More importantly, we prepared this report of an extremely rare problem anesthesiologists may encounter with the assistance of ChatGPT, which is a large language model capable of generating fluently composed text on a multitude of topics, even one as obscure as JHF.

The condition was first characterized by Murray in 1873, and at that time was known as molloscum fibrosum [11]. It is exceedingly rare, with only a few hundred cases reported worldwide, mostly describing the known hyaline fibromatosis syndrome (HFS) management of individual patients. It is properly understood to be part of a spectrum of related conditions known as HFS, comprising JHF and a more severe form presenting in infancy, infantile systemic hyalinosis (ISH) [12]. Both conditions are related to mutations in chromosome 4q21 in the anthrax toxin receptor-2 gene (ANTXR2, also known as capillary morphogenesis gene 2 [CMG2]) [13]. It appears that the types of mutations in the more severe ISH are more extensive and involve an extracellular domain, while those in JHF tend to be in-frame (missense) mutations of the cytosolic domain [14]. The normal physiologic function of ANTXR2 is not fully characterized, but the protein binds collagen IV and laminin, suggesting that it may be involved in extracellular matrix adhesion. Defects in this molecule lead to abnormal deposition of hyaline material in perivascular areas, presenting as cutaneous lesions, joint contractures, and in some cases internal organ dysfunction. In ISH disruptions to bone and internal organs are seen and often lead to death in childhood, whereas the course of JHF is often much more benign and tends to involve primarily skin and joints [12,13]. The mutation is inherited in an autosomal recessive fashion and has been reported somewhat more frequently from areas of the world in which consanguineous parentage is more common (Middle East, North Africa) than in the West [15,16].

The obscurity of the condition is illustrated by no entry for it in UpToDate, a comprehensive, peer-reviewed, continuously updated online medical reference [17]. It is also not mentioned in the text Anesthesia and Uncommon Diseases or any major English language anesthesia textbook. Unsurprisingly, there are only a handful of reports of anesthetic management of JHF or ISH, and most commonly in young children [2-10]. Two reports of anesthetic management of adults with HFS have also been published [4,5].

The anesthetic implications of HFS are primarily related to patient positioning and airway management. The presence of flexion contractures and large cutaneous lesions may complicate patient positioning for intravenous line placement, anesthesia induction, regional anesthesia procedures, or the surgery itself [3,4,10,13,16,18]. Our patient did not manifest any such limitations, illustrating the variable presentation of the disease. Particularly in pediatric patients with severe ISH, airway management can be very complicated. Gingival hyperplasia, hyaline infiltration of the tongue, and cutaneous lesions around the lips may be so extreme that insertion of conventional or video laryngoscopes may be impossible. Even if insertion of a laryngoscope is feasible, temporomandibular joint contracture and neck immobility may complicate visualization of the larynx. Many case reports have described use of a flexible bronchoscope to secure the airway [3,4,6,7]. In our patient, the airway appeared normal, and she reported no prior problems with anesthesia. Given the short expected surgical duration, we elected to use a supraglottic airway, which was positioned easily. We believe this to be the only report of the use of a supraglottic airway (in this case an LMA) in HFS. We suggest that such an approach may be reasonable to consider for cases involving straightforward cutaneous resections even if the airway appears compromised, as a flexible bronchoscope could be placed through the LMA should endotracheal intubation become necessary. In general, drug responses once anesthetized have reportedly been normal, though one case of apparent succinylcholine resistance in a 47-year-old patient was reported, but of uncertain relationship to JHF [5]. Similarly, our patient reacted predictably to intravenous and inhalation anesthetics, requiring normal to slightly greater than expected dosages.

This case report was written with the assistance of ChatGPT in response to a challenge issued by the journal [19]. ChatGPT is a large language model that has been trained using deep learning techniques by OpenAI. It is a type of artificial intelligence system that has the ability to generate fluent language on a multitude of topics, including those related to the biomedical field. One of the unique features of ChatGPT is its ability to "predict the next word" in a given sentence or text, which allows it to generate coherent and natural-sounding language. This is achieved by training the model on large amounts of text data, which allows it to learn the patterns and structures of language. As a result, ChatGPT is able to generate language that is both grammatically correct and semantically meaningful, making it a valuable tool for natural language processing in the biomedical field.

**Figure 3:**
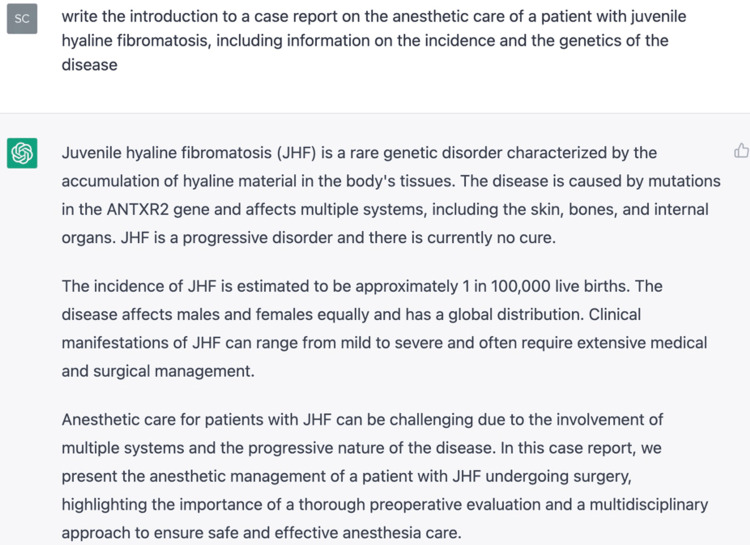
ChatGPT-drafted introduction of this report

**Figure 4:**
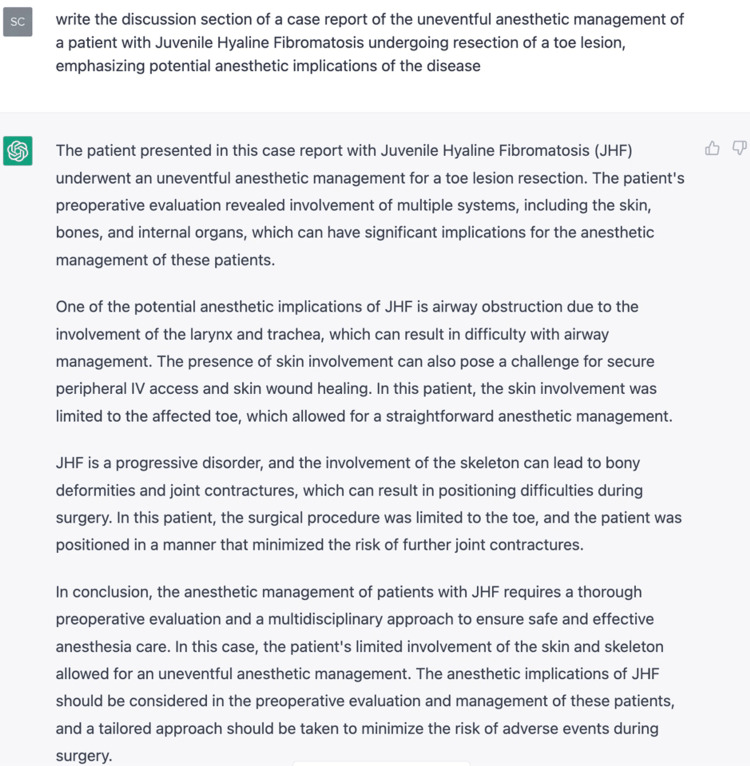
ChatGPT draft of the conclusion section of this report

**Figure 5:**
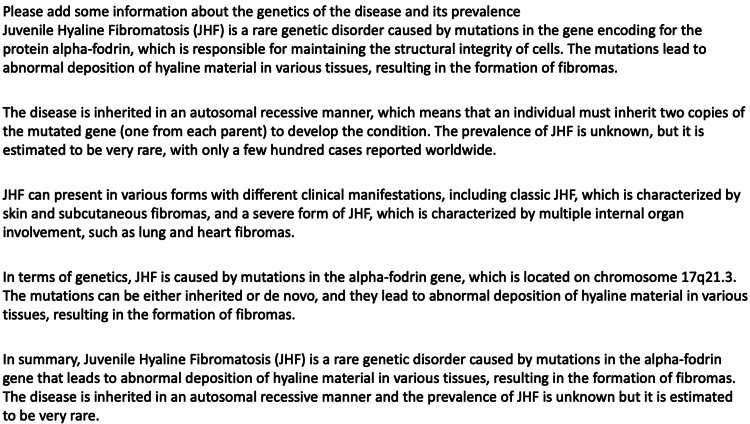
ChatGPT’s original erroneous description of the genetics of the disease

The preceding paragraph following the first sentence was written by ChatGPT, as was the introduction to this report and the conclusion which follows (Figures 3-5). It is important to note that the software could never actually write the case presentation (on which it could not have been trained), nor could it likely meaningfully compare and contrast this case to others in the literature. Indeed, when the software was initially asked to "write a case report on the anesthetic management of JHF" it composed an entirely fictitious case about the management of a 13-year-old boy presenting airway difficulty during anesthesia. While it may have done so by modeling its prose after previously published case reports, it was obviously unsuited to drafting the full report of an actual case. Therefore, we asked it to draft only certain sections, such as the introduction and parts of the discussion.

Moreover, the exercise of composing this report also illustrates several of the other challenges of using large language models in creative or scholarly writing. Though trained on a huge data set and refined by human input (“reinforcement learning”) and ultimately through a separate “reward” AI model tuning the output of the main language model (Proximal Policy Optimization), ChatGPT nonetheless has no semantic understanding of the text it writes [20]. This can lead it to generate fluent but erroneous prose. For example, when first queried for assistance with this report, the system eloquently but incorrectly attributed JHF to a mutation in the alpha-fodrin gene, which is located on chromosome 17q21.3 (Figure 5). In addition, it significantly overestimated the incidence of the condition in the introduction (1:100,000 live births-this figure was redacted from the final version), as this would imply 40 cases per year in the U.S. and 1400 cases per year worldwide, far more than is apparent from the literature. Moreover, when prompted to “please cite several references from the literature to accompany a case report on the anesthetic implications of Juvenile Hyaline Fibromatosis” the system returned 5 citations, all from legitimate anesthesiology journals, complete with digital object identifiers, none of which were familiar to the authors. Further investigations demonstrated that all five were entirely fictitious. Finally, the repeated queries and fact checking required to ensure the veracity of the finished product added significantly to the task of preparing this paper. Thus, we suggest that any use of ChatGPT at this stage of its evolution be done in the role of generating a rough draft or “head start” on a paper, but expert review and refinement is essential.

## Conclusions

We conclude by offering two versions of the summary of our work. 

First, per ChatGPT: In conclusion, the anesthetic management of patients with JHF requires a thorough preoperative evaluation and a multidisciplinary approach to ensure safe and effective anesthesia care. In this case, the patient's limited involvement of the skin and skeleton allowed for uneventful anesthetic management. The anesthetic implications of JHF should be considered in the preoperative evaluation and management of these patients, and a tailored approach should be taken to minimize the risk of adverse events during surgery.

Second, per the authors: ChatGPT is a powerful text composition tool that offers the possibility of simplifying or speeding up some writing tasks, including those in the biomedical literature. However, its limitations are significant, including the possibility of drafting entirely incorrect prose, so careful human diligence in using the tool remains essential.
